# Income inequality in uptake of voluntary versus organised breast cancer screening: evidence from the British Household Panel Survey

**DOI:** 10.1186/s12889-018-5139-9

**Published:** 2018-02-14

**Authors:** Patricia Carney, Ciaran O’Neill

**Affiliations:** 1 Centre for Economic and Social Research on Dementia, National University of Ireland Galway, Galway, Ireland; 2 Centre for Public Health, Queens University Belfast, Belfast, UK

**Keywords:** Health economics, Breast screening, Income inequality

## Abstract

**Background:**

This paper measures income-related inequality in uptake of breast cancer screening among women before and after a policy change to extend the screening programme to women aged 65 to 70. Prior to programme expansion women aged 50 to 64 were invited for screening under the national cancer screening programme in England and Wales whereas women in the 65 to 70 age cohort could elect to be screened by personally organising a screen. This will give a deeper insight into the nature of inequality in screening and the impact of policies aimed at widening the access related to age on inequality of uptake.

**Methods:**

Taking advantage of this natural experiment, inequality is quantified across the different age cohorts and time periods with the use of concentration indices (CI). Using data from the British Household Panel Survey, information on screening attendance, equivalised household income and age was taken for the three years prior to the programme expansion and the three years immediately following the policy change.

**Results:**

Results show that following the expansion, inequality significantly reduced for the 50-64 age group, prior to the expansion there was a pro-rich inequality in screening uptake. There is also evidence of a reduction in income inequality in screening uptake among those aged 65 to 70 and an increase in the number of women attending screening from this older age cohort.

**Conclusions:**

This indicates that an organised breast screening programme is likely to reduce income related inequality over a screening programme where women must organise their own screen. This is important when breast screening is one of the main methods used to detect breast cancer at an earlier stage which improves outcomes for women and reduces treatment costs.

## Background

The effectiveness and cost-effectiveness of breast cancer screening has been the subject of debate in recent years [1–3]. It is argued that, while it is likely to reduce mortality it is likely to increase morbidity as a result of over-diagnosis. Cost-effectiveness varies depending on a range of factors including assumptions regarding the extent to which screening advances the diagnosis of cancer and the reduction in cancer incidence after screening stops. On balance, evidence appears to support the continued operation of population based breast cancer screening among specific age groups based on their relative risk and capacity to benefit [4]. The debate over the value of screening [4–7] however, draws attention to the sometimes subtle arguments as well as uncertainties required in assessments of the value of screening among well-informed independent observers.

Among the public the challenges for informed decision making are likely to be even greater and it is unclear what, if any, impact discussion in the literature on the evidence has on individual decisions of whether or not to screen. Studies have shown that over half (56%) of breast cancers detected in Britain in 2007 were screen detected, indicating the programme is successful in engaging women in the relevant age range to screen [8]. The decision of whether or not to screen however, remains the choice of the individual, a choice that requires the individual to process the information they are presented with, including that from the cancer screening programme. The cost of processing and acting on such information, related to factors such as education [9] and the opportunity costs of time [10], is unlikely to be distributed equally across the population. In consequence a socio-economic gradient might be expected to exist with those who face greater transaction costs being less likely to avail of screening.

In Britain, an extension of the programme to include older women was introduced in 2001, contributing to a reduction in such barriers among the age group concerned. However, it is unclear whether this would prompt a disproportionate response among those of lower socio-economic status, for whom transaction costs related to organising and availing of a screen might have been relatively highest, or those from higher socio-economic groups for whom the cost of processing information regarding the value of a screen might have been relatively low. In consequence one would reasonably expect any socio-economic gradient prior to the policy change to be impacted by the expansion of the programme, though it is not immediately obvious in which direction the impact might operate. Such an assessment is clearly important in assessing the success, or otherwise, of a policy intervention in terms of its impact on equity. That the expansion of the programme and attendant publicity might also be expected to have spill-over effects among younger women in terms of the socio-economic profile of women attending also exists and is worthy of investigation. If peer support contributes to uptake decisions, a policy that differentially benefits those of a particular socio-economic status might be reasonably expected to attenuate any socio-economic gradient among other younger women.

In this paper we estimate and compare the socio-economic gradient in respect of uptake of breast cancer screening in Britain before and after an extension of the eligible age range examining women directly and potentially indirectly affected.

### Data

The variables presented in this study are based on data from the British Household Panel Survey (BHPS) carried out in Britain from September 1990-August 2008. The survey was carried out from September of each year based with questions relating to the previous 12 months. The BHPS dataset consists of approximately 10,000 interviewed individuals in total, on approximately 5500 households drawn from across Britain, using a stratified random sampling approach [11]. In this analysis, the screening behaviour of two sub-samples were examined and compared.

Breast cancer screening is recommended for women aged 50 to 70 in the UK. In some areas in the UK women aged 47 to 49 and 71 to 73 receive invitations for screening. This is part of a trial to decide if the screening age should be extended to include women in these age categories. This is not accounted for in this study. From programme inception in 1990 until 2000 women aged 50-64 were invited for screening free of charge every three years, with the programme being extended to include women aged 65 to 70 in 2001 in England and Wales and in 2003 in Scotland [12]. It was announced in 2009 that Northern Ireland was to follow with the expansion of the BSP to include women aged 65-70.

## Methods

Data were extracted from the BHPS for all women present in the sample in England and Wales from September 1999 to August 2002, and again all women present from September 2005 to August 2008. Women aged 53 to 70 in two time periods were studied; the first represents screening patterns prior to the expansion of the screening programme and the second time period is after the policy change regarding the eligible age range for screening.

Breast screening is offered on a 3 year cycle commencing at age 50. To capture exposure to screening women aged 53 to 70 were therefore considered to constitute the age offered screening. This group were separated into those aged 53 to 64 and 65 to 70, constituting the group to whom screens were offered throughout and those to whom screening was extended respectively.

Equivalised household income (gross) averaged over two, three year periods was used as the ranking variable in the construction of the concentration indices (CI). McClements equivalisation scale was used to divide total household income which applies a weighting for each individual in the household. The first adult is accorded a weight of 0.61, spouse 0.39, other second adult 0.46, third adult 0.42, and further adult 0.36. Children are allocated a weighting depending on their age, 0-1 years is 0.09, 2-4 years 0.18, 5-7 years 0.21, 8-10 years is 0.23, 11-12 0.25, 13-15 years 0.27 or 16+ years 0.36. Income is recorded as a continuous variable in the BHPS. Winzorised equivalised household income was used as the ranking variable. By winzorising the outliers are replaced with the average of the percentile as oppose to trimming them away. Income was chosen rather than, for example, education or employment status as it offered more ranking points across which to estimate a CI. Equivalised household income was chosen to take account of total household income and calls upon this rather than simply that earned by the individual, this gives a better indication of socio-economic status. A binary variable is created for women that reported having screened at least once in the three year window observed. The data for screening in each of the three year periods is pooled.

Data were analysed using a CI in which both Wagstaff and Erreygers corrections to take account of the dichotomous nature of screening uptake (screened in the past three years yes/no) were estimated [13, 14].

The health CI, as first introduced by Kakwani et al. [15], Wagstaff et al. [16] and Wagstaff [13], is a summary index of socio-economic related health inequality. The index is constructed so as to be bounded between + 1 and − 1; a negative value is recorded if the health measure of interest is disproportionately concentrated amongst the poor relative to their representation in the population and positive if concentrated amongst the rich. The CI measures health inequality by using income as its ranking variable and the health measure, in this case attendance at breast screening, as the dependent variable [17], thus calculating how screening is distributed proportionately across the socio-economic ranking variable, in this case equivalised household income. Socio-economic related health inequality, in this instance, refers to variation in uptake of breast screening among the age group clinically recommended as you move along the distribution of socio-economic status. The main condition defining a CI is that it is based on rank ordering based solely on the ranking variable.

The CI is contained in eq. (1).

1 $$ CI=\frac{2}{n{\mu}_h}\sum \limits_{i=1}^n{h}_i{R}_i-1 $$

*N* is population size, *h*_*i*_ is health (whether the individual availed of breast cancer screening), *μ*_*h*_is the mean of the dependent variable (proportion who uptake screening), *R*_*i*_ = (*λ*_*i*_*/n*) is fractional rank (λ_i_) with respect to the socio-economic distribution, λ*i* = 1 (poorest), λ_n_ = n (richest). Kakwani et al. [15] illustrated that the standard error for the CI, illustrated above, can be calculated using the following eq. (2) to estimate the variance:

2 $$ \mathit{\operatorname{var}}(CI)=\frac{1}{n}\left(\frac{1}{n}\sum \limits_{i=1}^n{a}_i^2-{\left(1- CI\right)}^2\right) $$

The health variable is assumed to be continuous in the case of the CI, for example childhood height. However, if the variable being examined is binary, such as if the woman has gone for a breast screen or not in the previous three years, the mean of the distribution places limits on the possible bounds of the CI and therefore the traditional CI is not bounded between − 1 and + 1. Consequently an adjusted index must be used.

Corrections to the CI in the case of dichotomous variables have been suggested by Wagstaff [13] and Errygers [14]. Debate has continued in the literature as to which is the more appropriate correction to use when examining relative inequality [18].

### Normalised Concentration Indices

Wagstaff [13] and Eyyergers’ [14] suggested amending the traditional CI, as presented above, in order to correct the bias caused by bounded variables. The correction suggested by Wagstaff, is outlined in eq. 3 and contains three of the four properties of rank dependent indices as suggested by Kjellsson and Gerdtham [18], the mirror property, transfer and cardinal invariance. The mirror property refers to the measuring of inequality in both directions and getting the same index measure. Put simply it means that if you change the value of the bounded variable around, for example assign a value of 1 to women that don’t screen and 0 to women that do, then the index would be the same size but have the opposite sign.

Wagstaff’s CI is defined as:

3 $$ {Cl}_W=\frac{\mu_h\left({b}_h-{a}_h\right)}{\left({b}_h-{\mu}_h\right)\left({\mu}_{h-}{a}_h\right)}\left(\frac{2}{n{\mu}_h}\sum \limits_{i=1}^n{h}_i{R}_i-1\right) $$

Also expressed as:

4 $$ {CI}_w=\frac{CI}{1-{\mu}_h} $$

The transfer condition is that the concentration recognises a small transfer of ‘health’ from richer to poorer as a pro-poor change in the index. Scale invariance is when the index is not dependent upon the linear formation of the health variable, so for example if screening was measured in months or years, this scale invariance condition allows us to calculate a CI to measure if attendance is most concentrated among the rich or poor. Kjellsson and Gerdtham [18] also acknowledge one other property of some rank dependent inequality indices not met by the Wagstaff index, level independence. They describe this as when “an equal increment of health for all individuals does not affect the index, that is the index is invariant to scalar addition even when the bounds of the variable are kept constant.” This is addressed in the CI correction developed by Erregyers.

5 $$ {CI}_E=\frac{4{\mu}_h}{\left({b}_h-{a}_h\right)}\left(\frac{2}{n{\mu}_h}\sum \limits_{i=1}^n{h}_i{R}_i-1\right) $$

*b*_*h*_ is the upper bound, *a*_*h*_ the lower bound, *R*_*i*_ is the weighted fractional rank of individual i, *h*_*i*_ is the health indicator for individual i and *nμ*_*h*_is the proportion of the population that attend screening in each three year time period.

The main relevant differences between the two normalised indices are they report different types of inequality; Erreygers’ index measures absolute inequality and Wagstaff’s relative inequality.

The second difference between both indices is that Wagstaff’s index is bounded by − 1 and + 1, whereas Erreygers’ index is bounded by

6 $$ -\frac{\left({b}_h-{\mu}_h\right)\left({\mu}_h-{a}_h\right)}{\mu_h\left({b}_h-{a}_h\right)}\le {CI}_E\ge +\frac{\left({b}_h-{\mu}_h\right)\left({\mu}_h-{a}_h\right)}{\mu_h\left({b}_h-{a}_h\right)} $$

In this paper we have estimated both Wagstaff and Erryger’s corrected CIs using the conindex Stata command written by O’Donnell et al. (2016) [19].

## Results

Table 1 contains descriptive statistics for the women included in this study for the two time periods, namely 1999-2002 and 2005-2008.

**Table 1:** Sample Descriptive Statistics for the two time periods

Variable	Pre Sample characteristics		Post Sample characteristics	
	Age 53-64 *N =* 983	Age 53-64 *N =* 872	Age 65-70 *N =* 365	Age 65-70 *N =* 294
	Mean (SD)	Mean (SD)	Mean (SD)	Mean (SD)
Equivalised Household Income	£23,877 (£16,638)	£18,706 (£11,235)	£29,595 (£18,202)	£22,183 (£9690)
Number Earners in Household	1.54 (1.08)	1.53 (1.12)	0.21 (0.50)	0.28 (0.60)
Number dependants under 16	0.045 (0.26)	0.056(0.31)	0.03 (0.25)	0.02 (0.18)
Age	58 (3.16)	59 (3.08)	68 (1.68)	67 (1.74)
Proportion that attend breast screening	72%	69%	37%	47%
Higher Level Education	13%	18%	12%	12%
Secondary Level Education	32%	39%	25%	26%
Primary Level Education	55%	43%	63%	62%
Ten or more GP Visits	13%	11%	13%	12%
6 to 10 GP Visits	14%	13%	14%	15%
3 to 5 GP Visits	21%	22%	30%	23%
1 or 2 GP Visits	33%	32%	29%	35%
0 GP Visits	19%	22%	14%	15%
Smokers	25%	20%	16%	13%
Self-reported health status – Excellent	20%	21%	17%	15%
Self-reported Health Status – Good	43%	44%	43%	45%
Self-reported health status – Fair	24%	24%	28%	27%
Self-reported health status – Poor	10%	8%	8%	11%
Self-Reported Health Status- Very Poor	3%	3%	3%	2%
Marital Status – Married	72%	72%	63%	67%
Marital Status – Separated	1%	1%	0%	1%
Marital Status – Divorced	14%	16%	12%	12%
Marital Status – Widowed	9%	7%	19%	18%
Marital Status – Never Married	4%	4%	6%	2%

SD = Standard Deviation

As seen in Table 1 average equivalised household income has increased in the second time period; this is not surprising as it has not been adjusted for inflation. There is a marginally higher average number of earners per household in the latter time period, which may have an impact on screening attendance. In the second time period education rates are higher, and slightly fewer women visit their GP. Fewer in the sample smoke in the second period and little is changed in self-reported health and marital status.

Following the expansion breast screening attendance increased from 37% to 47% within the older cohort included in this study. Among the younger group screening attendance in the first time period was 72%, this reduced slightly to 69% post expansion. Interestingly in the second time period a marginally smaller proportion of the sample attend screening in the 53-64 age categories. A higher proportion of the older women attend in the second time period than in the first which is as one might expect given the expansion of the programme to include women in this age category. A t-test of the difference in proportions supported the contention that there had been an increase in the proportion of older women in the eligible age range availing of screening over time.

**Fig. 1:**
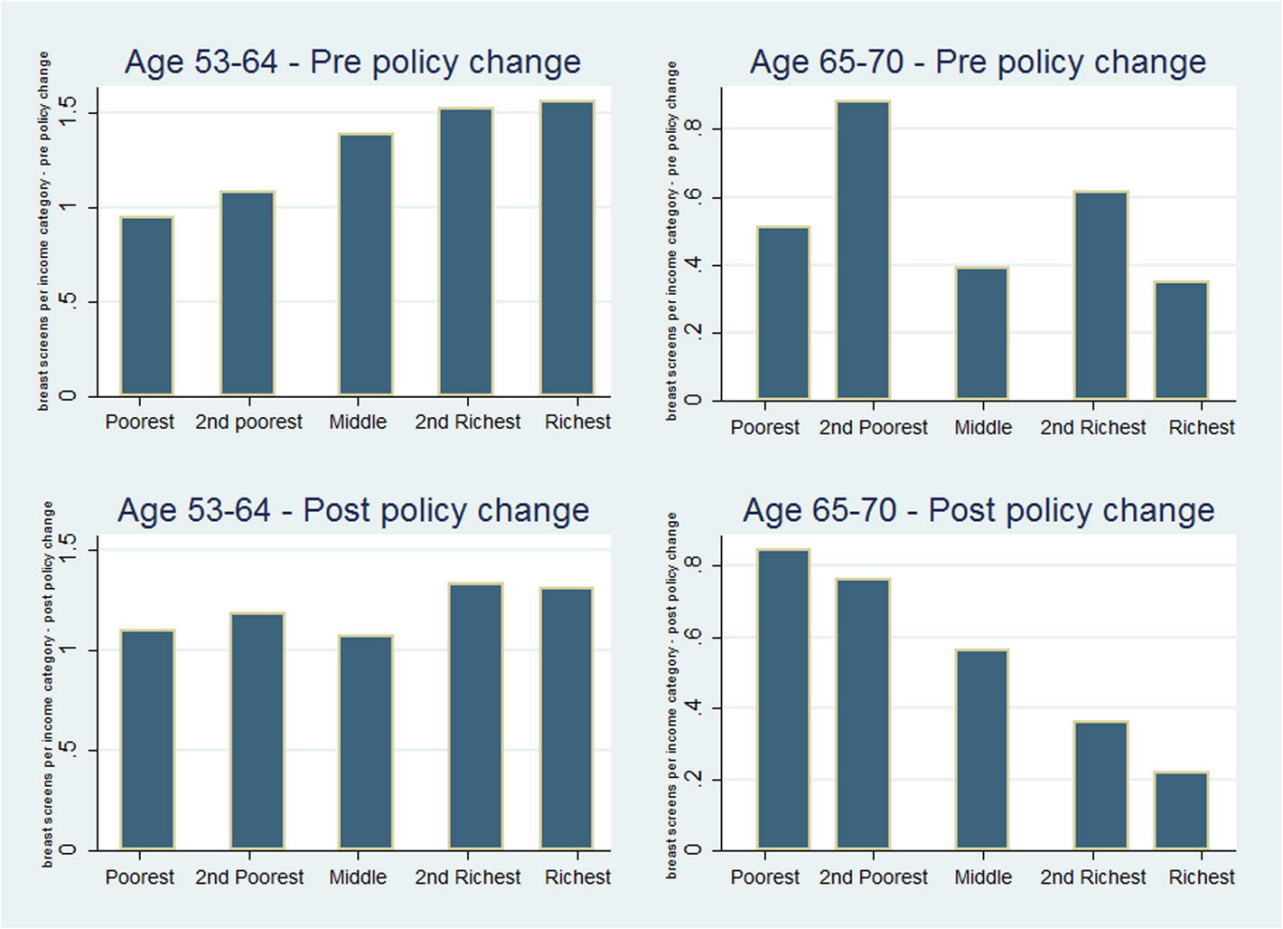
Equivalised household income distribution for the four samples

Figure 1 illustrates breast cancer screening uptake distributed across equivalised household income quintiles. In both time periods, a higher proportion of women in the richest income quintiles attend at least one screen in the three year time period. To give greater focus to our study and avoid the potential for confounding associated with different behaviours across age groups we compare CIs for those aged 53-64 before the policy change only with those 53-64 after the policy change. Likewise we compare CIs for those aged 65-70 before the policy change only with those in this age group afterwards.

**Fig. 2:**
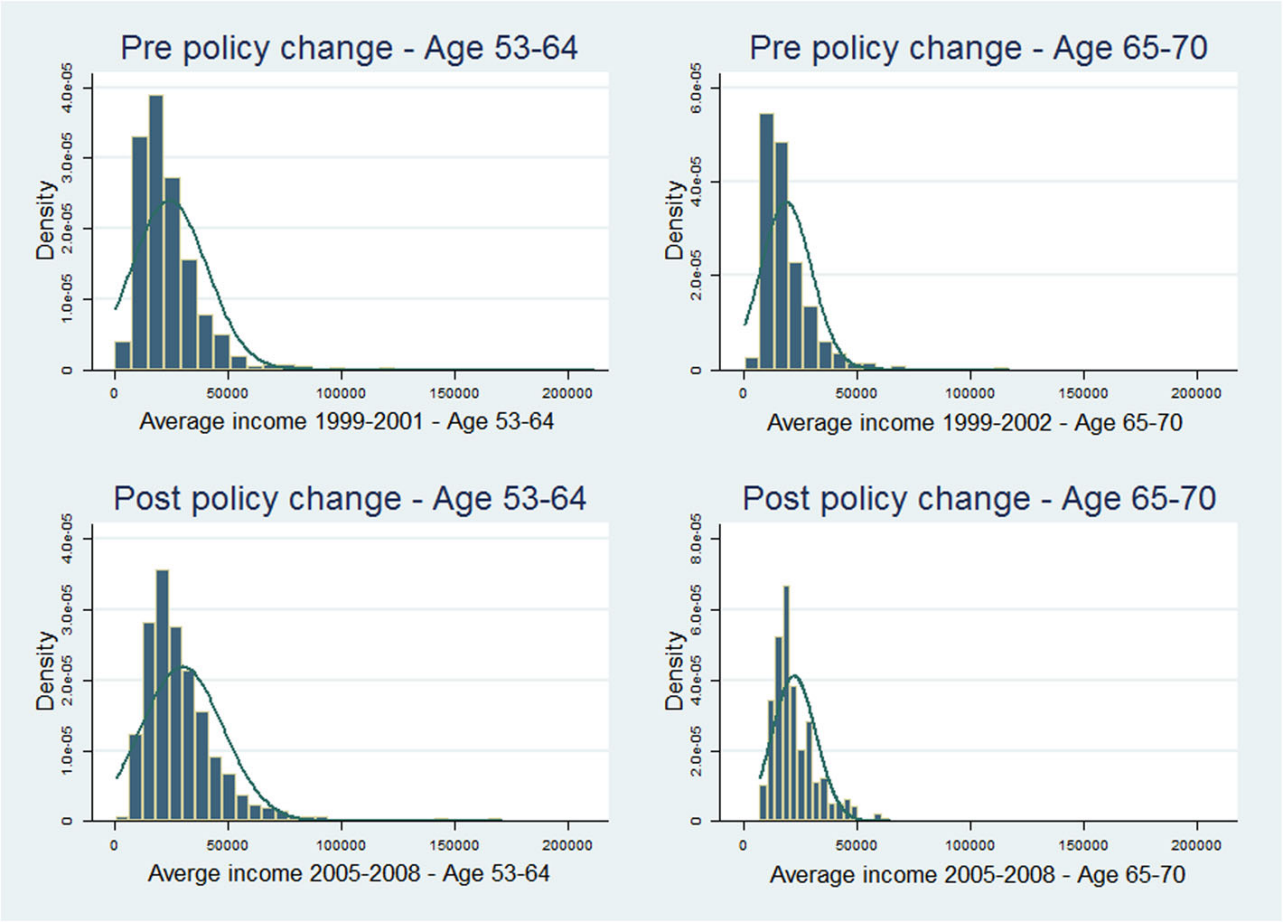
Screening uptake by income quintile

Figure 2 above illustrates screening uptake in each income quartile. Prior to the policy change screening attendance is lower among the poorest age categories though among older women a less obvious relationship with income is apparent. After the policy change a more evident pro poor pattern of uptake among the over 65 age category is evident, while among those aged 53-64 a more equitable pattern of uptake is evident.

In Fig. 1 income distributions are presented for both age groups before and after the policy change. The income distributions are more concentrated in the lower end of the scale for all age groups; however the skewness appears smaller for the older age category, the 65-70 age group. This is confirmed by the figures in Table 1 where skewness and the comparison of the mean relative to the median as well as the standard deviation for older age cohorts relative to younger cohorts show the more even distribution of income.

As can be seen in Table 2 the CI results indicate that in the wake of the policy change less pro-rich patterns of uptake are evident in both age groups.

**Table 2:** Concentration Indices

	Wagstaff’s Index (Standard Error)	Erreygers’ Index (Standard Error)
Pre policy Change England and Wales Women Aged 53-64 (*N* = 966)	0.097* (0.04)	0.078* (0.032)
Pre Policy Change England and Wales Women Aged 65-70 (*N* = 359)	0.083 (0.063)	0.078 (0.059)
Post Policy Change England and Wales Women Aged 53-64 (N = 872)	0.008 (0.042)	0.007 (0.036)
Post Policy Change England and Wales Women Aged 65-70 (N = 294)	−0.03 (0.068)	−0.03 (0.068)

* Statistically significant at 95% level

## Discussion

Among women aged 65-70 while income inequality fell in the wake of the programme expansion neither the level of inequality before the policy change nor in its aftermath was statistically significant. Perhaps surprisingly, given they were not the direct target of the policy, among women aged 53-64 the pro-rich pattern of uptake prior to the policy change did change with no statistically significant gradient in uptake being evident after the introduction of the change.

The socio-economic gradients and changes in these in the wake of the policy change are not readily interpretable by reference to transaction costs or financial barriers and how these are distributed across the population. A reduction in transaction costs associated with organizing a screen and of the financial barriers of paying for a screen would be expected to disproportionately impact on those who were the target of the policy change – those aged 65-70 – and among this group among those with lower incomes for whom barriers would be most evident in particular. While the degree of pro-rich inequality is attenuated, changes in the socio-economic gradient are negligible. By contrast, among younger women, where a marked pro-rich pattern of uptake was evident before the policy change, no such gradient is evident in its wake. Why this should be the case is not entirely clear. Publicity associated with the extension of the eligible age range may have contributed to relatively greater uptake among less well-off younger women though this seems unlikely. Greater uptake among less well-off older women may have encouraged greater uptake among less well-off younger women with whom they are familiar. Such “endorsement” may be particularly important among those for whom transaction costs are high or among whom health literacy is low. It is also conceivable that the observed changes with respect to younger women are unrelated to the policy change and rather reflect a distinct trend of greater engagement among less well-off younger women or a response to other initiatives. The changing pattern of uptake does point to the potential existence of “spill-over” effects though it is unclear as to what mechanism might be at work.

Some caution is warranted in our interpretation of results. The comparison of uptake before and after the policy change offers only a crude mechanism by which to assess its effect. While in principle extending the analysis to Scotland – which experienced the policy change later than England and Wales – might offer further evidence, in practice small sample sizes (just 86 women aged 65-70 in Scotland in BHPS post policy change, for example), as well as differences in the overall context in which screening is offered, call into question the merit of such comparison. More broadly, the sample sizes for England and Wales underscore the importance of caution in drawing conclusions from the study. While overall the proportion of screening uptake rose and the socio-economic gradient in uptake fell in the aftermath of the policy change, caution is warranted in hailing this as a success given differing income distributions between the two age groups. Moreover, it is important to note that we can say nothing from this analysis as to whether women (and society) benefit from the expansion in terms of morbidity and mortality reduction.

## Conclusion

In the wake of a policy change that extended access to publicly funded screening, the degree of pro-rich disparities in uptake fell among age groups covered and uncovered by the policy change. The reason underlying the change in behaviour appear to extend beyond financial barriers related to uptake of screening and suggest further research in this area may well prove useful in developing policies aimed at increasing uptake where appropriate. Following the expansion of the programme uptake and greater equity of uptake emerged which might be considered to provide evidence of its success. However, why the spill-over effects related to equity arise is unclear, suggesting some caution is needed hailing the success too strongly and that further research on its impact is warranted.
